# Replication of a whole school ethos-changing intervention: different context, similar effects, additional insights

**DOI:** 10.1186/s12889-015-1538-3

**Published:** 2015-03-19

**Authors:** Penelope Hawe, Lyndal Bond, Laura M Ghali, Rosemary Perry, Colleen M Davison, David M Casey, Helen Butler, Cynthia M Webster, Bert Scholz

**Affiliations:** O’Brien Institute of Public Health, University of Calgary, 3280 Hospital Drive NW, Calgary, AB T2N 4N6 Canada; The Australian Prevention Partnership Centre and Menzies Centre for Health Policy, University of Sydney, Sydney, NSW 2006 Australia; Centre for Excellence in Intervention and Prevention Science, 15-30 Pelham Street, Carlton, VIC 3053 Australia; The Ability Hub, 3rd Floor, 3820-24th Ave NW, Calgary, AB T3B 2X9 Canada; Department of Public Health Sciences, Queens University, Carruthers Hall, Office 203, 62 Fifth Field Company Lane, Kingston, ON K7L 3N6 Canada; Alberta Health Services, Centre 15, 1509 Centre Street SW, Calgary, AB T2G 2E6 Canada; Faculty of Education and Arts, Melbourne Campus (St Patricks), Australian Catholic University, Mary Glowrey Building, 115 Victoria Parade, Fitzroy, VIC 3065 Australia; Department of Marketing and Management, Macquarie University, Sydney, NSW 2109 Australia; Brooks Composite High School, Box 849, 650 - 4th Avenue, Brooks, AB T1R 0Z4 Canada

**Keywords:** Context, Complex intervention, Intervention theory, Risk behaviours, Whole school mental health, Adolescent health

## Abstract

**Background:**

Whole school, ethos-changing interventions reduce risk behaviours in middle adolescence, more than curriculum-based approaches. Effects on older ages are not known. We set out to replicate one of these interventions, Australia’s Gatehouse Project, in a rural Canadian high school.

**Methods:**

A guided, whole school change process sought to make students feel more safe, connected, and valued by: changes in teaching practices, orientation processes, professional development of staff, recognition and reward mechanisms, elevating student voice, and strategies to involve greater proactivity and participation. We conducted risk behaviour surveys in grades 10 to 12 before the intervention and 2 years afterwards, and social network analyses with the staff. Changes in health and health risk behaviours were assessed using chi-square. Interactions between the intervention and gender and between the intervention and school engagement were assessed using interaction terms in logistic regression models. Changes in the density of relationships among staff were tested with methods analogous to paired t-tests.

**Results:**

Like Gatehouse, there was no statistically significant reduction in depressive symptoms or bullying, though the trend was in that direction. Among girls, there was a statistically significant decrease in low school engagement (45% relative reduction), and decreases in drinking (46% relative reduction), unprotected sex (61% relative reduction) and poor health (relative reduction of 73%). The reduction in drinking matched the national trend. Reductions in unprotected sex and poor health went against the national trend. We found no statistically significant changes for boys. The effects coincided with statistically significant increases in the densities of staff networks, indicating that part of the mechanism may be through relationships at school.

**Conclusions:**

A non-specific, risk protective intervention in the social environment of the school had a significant impact on a cluster of risk behaviours for girls. Results were remarkably like reports from similar school environment interventions elsewhere, albeit with different behaviours being affected. It may be that this type of intervention activates change processes that interact highly with context, impacting different risks differently, according to the prevalence, salience and distribution of the risk and the interconnectivity of relationships between staff and students. This requires further exploration.

## Background

Behaviours commonly begun in adolescence, such as substance use and unsafe sex, can have immediate negative consequences for young people [1] as well as contributing to major health problems in adulthood [2-5]. A common first reaction to this scenario in schools is to develop curriculum-based health education programs to develop knowledge, skills and attitudes to overcome risky behaviours. But the effects of these types of programs have been minimal [6-9]. A further limitation of such an approach is that it does not recognise the importance of the social context in which these behaviours take place. Nor does it take advantage of the fact that many problems share a common risk process [10-13] with alienation and disengagement from school being a common predictor of mental health and substance abuse [14,15].

By contrast, whole school interventions take a social ecological approach to target the social environment of the school itself to build a more inclusive and emotionally supportive school ethos [16]. School ethos is the set of values, practices and attitudes that distinguish one school from another [17]. In Australia, the Gatehouse Project was a cluster randomised trial with 26 schools. It used a whole school ethos-changing approach achieving risk reductions in the order of 25-40% in substance use in a cohort of Grade 8 students in the intervention schools followed longitudinally two and three years after the intervention began (i.e., in Grades 9 and 10 at the time of assessment of outcomes) [18,19]. Similar effects were also shown in Grade 8 students assessed cross-sectionally four years after the intervention team began and two years after the intervention had formally ended [20].

The study described in this paper set out to replicate the Gatehouse Project in Canada. Specifically we wished to see if the effects would be similar with an older age group (Grades 10–12) and to determine if effects would be observable across a broader span of risk behaviours. The study in a single school was an opportunity for a new team to learn how to conduct whole school mental health promotion, prior to a larger program involving further replication and testing of the intervention in a cluster randomised trial.

### The Gatehouse Project in Australia

In line with the health promoting schools framework [21], the Gatehouse Project was a primary prevention program which included both institutional and individual focused components to promote the emotional and behavioural well-being of young people in secondary schools.

The Gatehouse Project drew on attachment and social support theories [22,23] and the Social Development Model – a theory which takes account of both risk factors and protective influences in young people’s community, school, family and peers [12,24,25]. The intervention was based on an understanding of risk processes for adolescent mental health that derive from social environments, such as isolation and alienation, as well as those that derive from an individual’s cognitive and social skills, such as dealing with common challenges and stresses [3]. The major objectives of the intervention were to increase levels of emotional well-being and hence reduce rates of substance abuse [4,5,26,27].

The two year intervention provided schools with a process for making changes to policies and practices across the whole school and to the social and learning environments of classrooms. It enabled teachers to guide students about the skills and knowledge for managing life’s every day challenges. There were no materials provided on substance use or risk behaviours. Strategies were used in subject areas determined by the schools, usually English or Health in Grade 8. In the later years of the project, teachers were encouraged to adapt and embed these strategies into their teaching [28]. Informed mainly from the education literature on school change [29] the design and implementation of the intervention was shaped to account for the school’s capacity to take on the intervention in terms of resources and pressures of competing priorities.

Schools developed strategies to promote connectedness through good communication, and a sense of security and positive regard through making students feel valued and included. Key elements were: the establishment and support of the school-based health action team; the identification of risk and protective factors in each school’s social and learning environment derived from local data to prompt action and prioritise actions; and identification of effective strategies to address these issues led by the school community [22]. A part-time facilitator provided professional development and ongoing support for the schools during the implementation of the intervention. The facilitators were experienced educators (former teachers). They provided professional development, guidance and resources and encouraged reflective practice. For a description of this role, information about the project logic, and examples of exercises and resources to encourage critical reflection on school processes, see Butler *et al.* [30].

### The CORE Connections Project in Canada

The Canadian replication study was devised in response to a request from a rural high school, via the local chief medical officer, and his concern about rising rates of sexually transmitted disease in the town. The school administration was advised that the research team had no specific capacity to address sexual health. But the results of the Gatehouse Project had been recently presented in Canada. What appealed to the school and the local health region representatives was its holistic approach, the emphasis on engagement, whole-school ethos, and the potential for wide benefits. Members of the research team travelled to Australia to gain some first-hand understanding from schools previously involved with the Gatehouse Project. The local name of the project, Creating Opportunity for Resilience and Engagement (CORE) captured the importance of an environment where students felt safe, valued, and connected. It was devised as a replication of the Gatehouse intervention in terms of Gatehouse being a process and set of strategies and principles, rather than the type of replication that might be expected if Gatehouse was a packaged program with set components to be conducted in certain ways on certain days [31].

The site was the only high school in a Canadian rural town of 11,500 people. The town had recently undergone a rapid population increase due to the expansion of the nearby meatpacking industry, although the number of enrolled students at the high school had remained relatively stable in the previous 10 year period [32]. The school comprised grades 10, 11 and 12. It had a traditional academic program as well as a vocational program including mechanics, construction, cosmetology, foods, visual and performing arts and information technology. The CORE project replicated all aspects of the Gatehouse process over a two year period: survey-feedback-action-survey with students; a school action team to identify priorities and select actions to improve the welcoming environment of the school (at a classroom, curriculum and whole school level); and professional development with teachers about emotional literacy. CORE began with assessment, feedback, consultation, and critical reflection about practices in the school that promoted emotional well-being and those that did not. A part-time facilitator (a retired primary school principal) guided the consultation and change process, coached by the research team. Later her role was taken over (part time) by a teacher and School Action team member, as CORE transitioned and was embedded into routine school practices. More information about the intervention is given in Table 1. Insights from the first of the photo voice projects, where students used cameras to reflect on the social environment of the school, are presented in Davison *et al*. [33].

**Table 1:** **CORE project intervention summary**

**Strategy objectives**			
• To increase awareness of the diverse experiences of the school climate or ethos from students, staff and teachers			
• To create opportunities for individual and collective actions to address identified needs and issues in all parts of the school and in association with the local community			
• To build capacity to address issues (i.e., commitment, skills, structures, resources, relationships) and create wide opportunities for engagement			
• To encourage evaluation, critical reflection and improved problem solving			
• To learn from failures and to build on strengths and successes			
**Design phases**			
1. Establishment. Form a steering group. Map existing activities, capacities and initiatives in the school			
2. Assessment. Pre-intervention baseline assessment of social climate and student health risks			
3. Design and implementation. Data feedback and priority setting, identify improvement strategies, develop professional development activities, implement policy and practice changes			
4. Post intervention evaluation. Assess students, staff and teachers. Plan for sustainability			
**ACTIONS**			
**Students**		**Staff and teachers**	**Community**
Photo voice methods to capture the views of different student groups about the school		Hold Professional Development (PD) at times when non-teaching staff can attend	Establishment of Community Liaison Committee. Meets monthly.
More student voice in decision-making-teacher and adviser student representatives to meet monthly with the principal		Mix up staff groups at meetings and events (seating). More events. Different formats. More people encouraged to take the lead roles.	Identify barriers and opportunities of students working part-time in local businesses. Interviews held with shops, businesses and youth group leaders.
Photo board at school to show more faces and different activities		Hold weekly PD 1 hour sessions, instead of eight full-day sessions year	Ideas for more youth-friendly workplaces, e.g., hours of work, time off in exams, time off for sports practice, etc.
New and better orientation practices for incoming Year 10		Establish new task group to address structural stressors (timetabling pressures)	Communication about CORE in local newspaper; events held with local Business Council
Poetry assignments in English class on the experience of being a teen		New regular Monday Memo from principal to shine the light on many people and connect staff and students with events	Regular column about CORE in the School newsletter and communications with parents
Logo competition to name CORE in the school		Appreciation board: recognition for good work done	
Consultation groups to identify student experience at the school		More walking the corridors and spaces where students say they feel unsafe	
Establishment of Teacher Advisor Period, so all students meet at the beginning of the day with one teacher		Picture board outside the staff room to show who-is-who on the staff	

Professional development was led by the CORE facilitator in the form of a weekly, one-hour session with all staff. In the second year of CORE, a visit to the school from the Gatehouse Project investigators enabled a two-day training session with school staff on whole school change and teaching for emotional well-being. This underlines an important point. The CORE intervention did not recommend the implementation of a particular curriculum package or set of lesson plans (which Gatehouse did). Rather, all teachers were encouraged to think about how to address emotional literacy in the regular curriculum, that is, in the teaching of Maths, English or Social Studies and to reflect on their pedagogy-how teaching styles may or may not promote well-being.

There was a further difference between CORE and Gatehouse. On hearing of the logic of kick-starting action by asking students about their connections and experience with school, the staff at the CORE school suggested that a similar process be undertaken with them. We felt this could strengthen the intervention. So CORE began with one-on-one qualitative consultation interviews with staff. The purpose was to identify current activities that promote social well-being at the school and to identify any issues that needed further addressing to improve the well-being of staff and students. The feedback of this data enabled priorities to be set.

Process monitoring of the CORE intervention was designed to assist with further replication and scale up. It consisted of journaling, focus groups and interviews. Journaling by the CORE facilitator recorded actions, events, strategies (summarised in Table 1) as well as thoughts, plans and documentation of professional development activities. This journal was used for ongoing review and discussion with the supervisor. Focus groups with students were conducted at the beginning of years 1 and 2 to interpret student data and guide further understanding of the social environment at the school and how it could be improved (4 groups, 4–6 students per group). Six interviews with four key informants gathered data on context, history, events, activities, staff and student reactions and how the activities were sustained.

## Methods

The study was set up to (1) investigate the association between disengagement with school and mental health and health risk behaviours; and (2) determine whether a whole school ethos-building intervention could improve mental health and risk behaviours. We used the same data collection instruments as the Gatehouse Project and added extra variables suited to older students (such as questions about unsafe sex) as well as general health status. The evaluation unfolded in two stages. First: the student surveys, conducted two years apart; and second: a social network survey of the staff, conducted after the results of both student surveys had been analysed. By “staff” we mean teachers as well as people in other occupational roles in the school such as librarian, teaching assistant and janitor. The surveys required active parental consent for student participation and consent by staff for their own participation, and was approved by the Conjoint Health Research Ethics Board of the University of Calgary.

### Data collection

#### Students

We administered the questionnaire to all students, whose parents had given consent, on a single day in June 2003 and June 2005. The survey was a paper and pencil questionnaire at the first administration and online at the second. The responses to the questionnaires were completely anonymous. Data from the Grade 10s was therefore not linked to the responses of the same students in Grade 12.

Mental health status was evaluated using the 13-item Short Mood and Feelings Questionnaire (SMFQ) [34]. Students were defined as having depressive symptoms if they scored 12 or more, reflecting a level of minor psychiatric morbidity at which a general practitioner might be concerned [35].

Tobacco, alcohol and marijuana use was measured by self-report following methods used by large-scale surveys across Canada. Participants were asked if they smoked (response options were no, non-smoker; no, ex-smoker, or yes). Smokers were asked how many days in the last week they smoked [36]. Regular smoking was defined as smoking on six or more days in the previous week. Participants were asked if they drank alcohol (yes or no). If yes, they were asked how often they drank with options being less than one day a month; less than one day a week; one day a week; 2 days a week; or 3 or more days a week. Regular drinking was defined as drinking on three or more days a week [37]. Participants were asked if they had ever used marijuana. Regular marijuana use was defined as using marijuana 1–2 days or more times a week in the previous six months [38].

Indicators of perceived availability of social attachments were adapted from the Interview Schedule for Social Interaction [39] for the Gatehouse Project [18,40]. Perceived availability of attachments means having someone to talk to, someone to depend on when angry or upset and having someone who can be trusted with private feelings and thoughts. Students were categorised as having good availability of attachments, poor availability or absent/very poor availability.

School climate was assessed using a 28-item scale. This scale was developed subsequent to the Gatehouse Project and drew on items used in the Gatehouse Project assessment of school connectedness and other scales relating to student perceptions of school [41-45]. The scale includes items about relationships with teachers (e.g., In this school teachers treat students with respect), school belonging (e.g., I feel I belong in this school), commitment to school (e.g., Doing well in school is important to me), and participation (e.g., Students have a say in decisions affecting them at this school).

Responses to the 28 items were summed to create a total score with a higher score indicating greater engagement. The internal reliability in the present study was high (Cronbach Alpha 0.93). Student Low school engagement was determined using the baseline data. Students were classified as having low school engagement if they scored in the lowest quartile (total score of <11). Further information about the items is available in Appendix.

Students were classified as bullied if they answered yes to any of four items addressing types of recent victimisation: namely being teased, having rumours spread about them, being deliberately excluded or experiencing physical threats or violence [46].

Sexual behaviour questions were drawn from Canada’s National Longitudinal Survey for Children and Youth [47]. Specifically, students were asked have they ever had sex and whether they used a condom the last time they had sexual intercourse.

Socio-demographic measures included family structure, language other than English spoken at home, country of birth, age and gender. The format of these questions was based on surveys used by Statistics Canada and Health Canada surveys.

#### Staff

The second part of the methods arose as an attempt to search for explanations for the possible mechanisms of action while the project was taking place. We conducted a social network analysis of the staff at the end of the first year of the project. Social network analysis is the study of the relationships or ties among “actors”, typically people or organisations. Social network analysis involves both the mapping the relationships or ties as well as calculating measures of network structure [48]. Frequently occurring exchanges among people over time establish a social pattern and build a social structure that influences things like information flow, how readily a particular person could rally resources or help, and how easy it might be to work collectively to tackle problems. In other words, social network analysis derives a picture of a social environment as a whole from the patterns of relationships and exchanges that occur within in it, rather than aggregating up or averaging individual self-judgements of a person’s connection, belonging, or sense of ease in that environment. If the social network analysis proved useful, we felt that it could expand to students in further development and testing of CORE.

We supplied a list of all staff in the school and asked staff to comment on three types of informal ties with each other (knowing by name; knowing more personally – defined as knowing personal information such as the name of a family member; and socialising outside of school) and two types of formal (professional role related) ties (engaging in regular conversations; and seeking advice in relation to a school matter). We measured relationships twice: at the end of the first year of the CORE Project and participants’ recollections of the existence of same relationships 12 months prior (when the CORE project began). The survey was conducted as a pen and paper survey, occupying 15 minutes at the beginning of a regularly scheduled professional development meeting. Questions were framed such that a respondent would answer that relationship was either present or absent (yes/no). In social network research, questions focused on usual transactions and routine relationships have been found to be more reliable than questions about events in a specific timeframe (such as the number of times in the last week) [49].

### Method of analysis

Prevalence estimates and 95% confidence intervals were estimated for the student health risk behaviours. Point estimates and 95% CIs were used to compare these prevalences across the two time periods using the chi square test statistic. Logistic regressions were undertaken to assess relative risk (reported as odds ratios) and to investigate interactions between the intervention and both gender and school engagement.

Analysis of the network survey was conducted using UCINET 6 [50]. Network graphs were drawn using Netdraw [51]. We calculated the density of each of the five relationships and tested the significance of changes in density between the beginning and end of the first year of CORE (12 months apart), using the bootstrap technique analogous to the classical paired t-tests for estimating the standard error of difference [52]. Density is the amount of ties that are present as a proportion of the total possible ties [53]. So if everyone knows each other the density score is 100%. We computed the two-step reach for all five relationships. Perhaps familiar as six degrees of separation, which is six-step reach, made known to many people by the popular play [54], two-step reach illustrates the proportion of the total number of people in the network who can be reached by a person within one link of the people who comprise his/her immediate ties. It is considered a measure of how quickly a person can mobilise resources or convey information to others. We did not expect all relationships to be reciprocal or two way. For example, Person A might recognise Person B, but not the other way around. However there were two relationships where we expected reciprocity. These were ‘regular conversations’ and ‘socialise with’. In these cases, to be conservative, we counted the relationship as being present only if both people said the tie was present. The technical term for this is symmetrising the data by the minimum value before proceeding to further analyses.

## Results

At baseline, 380 of 533 Grade 10, 11 and 12 students were surveyed, meaning that they had parental consent to take part and were present on the day (71% response rate). Two years later, of the 526 students in Grades 10, 11 and 12, 354 were present on the day with parental consent and completed the questionnaire (67% response rate).

Table 2 provides socio-demographic information about the students at baseline 2003 and two years later. At both times, the student demographics were very similar.

**Table 2:** **Sociodemographic characteristics of the students**

	**2003**		**2005**	
	**380**		**354**	
	**N**	**(%)**	**N**	**(%)**
**Male**	174	(45.9)	174	(49.2)
**Grade level**				
Grade 10	127	(33.4)	118	(33.3)
Grade 11	122	(32.1)	135	(38.1)
Grade 12	131	(34.5)	101	(28.5)
**Age**				
15 years or younger	61	(16.1)	69	(19.5)
16 years	106	(27.9)	112	(31.6)
17 years	140	(36.8)	113	(31.9)
18 years or older	73	(19.2)	60	(17.0)
**Country of birth**				
Canada	355	(93.9)	329	(92.9)
**Language spoken at home**				
English	343	(90.3)	318	(89.8)
Another language	4	(1.1)	6	(1.7)
English & another language	33	(8.7)	30	(8.5)
**Family structure**				
Lives with both parents	280	(73.7)	253	(71.5)
One parent & a stepparent	45	(11.8)	42	(11.9)
One parent only	41	(10.8)	49	(13.8)
Other	14	(3.7)	10	(2.8)

Table 3 shows the associations between school engagement and the health outcomes at baseline (2003). Students with lower engagement at school were more likely to have low social attachment, to have depressive symptoms, to be a smoker and to smoke regularly, to be regularly drinking and using marijuana and to rate their health as fair or poor.

**Table 3:** **Relationship between engagement with school, social attachment and health outcomes at baseline (unadjusted OR, 95% CI)**

	**Low engagement with school**		
	**OR**	**95% CI**	**P value**
**Bullied recently**	1.57	0.96, 2.56	0.073
**Low social attachment**	2.83	1.70, 4.70	<0.001
**Depressive symptoms**	2.71	1.48, 4.94	0.001
**Substance use**			
Smoker	3.70	1.99, 6.83	<0.001
Regular smoker	5.40	2.66, 10.94	<0.001
Drinker	1.38	0.81, 2.36	0.233
Regular drinker	3.49	1.03, 11.82	0.045
Regular marijuana	7.22	3.16, 16.51	<0.001
**Sexual activity**			
Unprotected sex last time	1.27	0.68, 2.40	0.455
**General health**			
Fair/poor	2.48	1.43, 4.28	0.001

We examined the possible interaction between school engagement and time for the health and risk behaviours and found a significant interaction for poor health. Of those who were most engaged with school, 15% had poor health in 2003 while in 2005 of those most engaged with school 5% reported poor health (OR 0.35, 95% CI: 0.19,0.63; p = 0.001). For those with low school engagement 31% and 29% reported poor health at 2003 and 2005 respectively.

We investigated changes in school engagement and the health and risk behaviours between baseline (2003) and two years later. As we found significant interaction effects between intervention and gender, Table 4 presents the prevalence estimates for 2003 and 2005, odds ratios, and 95% confidence intervals for boys and girls separately. The prevalence of most behaviours and poor health was higher for girls and boys at baseline. For girls, there was a reduction in all measures and these were statistically significant for low school engagement, drinking alcohol, unsafe sex and poor health. For boys there were small changes but no statistically significant effects. After the CORE intervention among the girls there was a 45% relative reduction in low school engagement; a 46% relative reduction in drinking alcohol, a 61% relative reduction in having unprotected sex and 73% relative reduction in those girls rating their health as fair or poor. The relative reduction is derived from the odds ratios. It is the difference between the odds ratio and one, expressed as a percentage.

**Table 4:** **The prevalence of victimisation, depressive symptoms and substance use before and after implementation of CORE**

	**Girls**					**Boys**				
	**2003**	**2005**				**2003**	**2005**			
	**206**	**180**				**174**	**174**			
	**%**	**%**	**OR**	**95% CI**	**p value**	**%**	**%**	**OR**	**95% CI**	**p value**
**Low school engagement**	24.4	15.0	0.55	0.33,0.92	0.022	25.9	29.9	1.22	0.76,1.95	0.403
**Low social attachment**	19.3	19.0	0.98	0.59, 1.63	0.938	32.8	29.7	0.87	0.55, 1.37	0.536
**Bullied recently**	59.9	54.2	0.79	0.52, 1.20	0.273	57.1	55.6	0.94	0.61, 1.46	0.793
**Depressive symptoms**	17.2	14.7	0.83	0.48, 1.44	0.512	11.2	11.3	1.01	0.52, 1.99	0.969
**Substance use**										
Smoker	13.4	9.6	0.68	0.36, 1.30	0.246	13.5	11.6	0.84	0.44, 1.60	0.596
Regular smoker	11.9	6.7	0.54	0.26, 1.11	0.088	7.6	5.8	0.75	0.32, 1.75	0.499
Drinker	73.4	59.8	0.54	0.35, 0.83	0.005	67.8	68.1	1.01	0.64, 1.59	0.967
Regular drinker	1.3	2.8	2.12	0.35,12.91	0.415	7.8	10.4	1.39	0.56, 3.43	0.481
Regular marijuana	21.8	15.6	0.66	0.29, 1.54	0.340	25.8	33.8	1.47	0.70, 3.08	0.305
**Sexual activity**										
Unprotected sex last time vs no sex or protected sex	18.1	8.0	0.39	0.20, 0.75	0.004	11.2	12.1	1.08	0.55, 2.11	0.818
**General health**										
Fair/poor	26.5	8.9	0.27	0.15, 0.50	<0.001	10.5	13.4	1.31	0.68, 2.53	0.417

We investigated grade level differences but found no statistically significant changes nor any patterns between the grades (results not shown).

Of the 53 teachers and staff at the school, 50 were present on the day of the survey and took part (94%). Table 5 illustrates the changes in the density of the staff and teacher relationships in the second year of the CORE intervention. The density of all networks increased in a 12 month period. Statistically significant changes were observed in all five relationships. Table 6 illustrates the change in the two-step reach of the principal, the vice principal as well as the five staff and teachers making up the CORE school action team. There were substantial improvements in the two-step reach. In terms of the advice seeking network in the school, for example, there were 10 people with 100% two-step reach at the end of CORE, whereas 12 months prior to this no-one had 100% two-step reach.

**Table 5:** **Density of staff and teacher relationships**

**RELATIONSHIP**	**2004**	**2005**	**Difference in density**	**t-statistic**	**P value**
	**Density %**	**Density %**	**%**		
**Recognise by name**	65.9	94.7	28.8	6.48	0.0002
**Socialise with**	5.9	7.8	1.9	2.52	0.0138
**Regular conversations with**	25.5	40.5	14.9	6.03	0.0002
**Know more personally**	29.0	38.6	9.8	6.26	0.0002
**Seek advice on a school matter**	15.2	20.7	5.5	4.62	0.0002

**Table 6:** **Two-step reach of key staff and teachers**

**RELATIONSHIP**	**2004**	**2005**
	**Two-step reach %**	**Two-step reach %**
**Recognise by name**		
Principal	100	100
Vice principal	100	100
SAT member 1	100	100
SAT member 2	100	100
SAT member 3	100	100
SAT member 4	100	100
SAT member 5	100	100
**Socialise with**		
Principal	27	33
Vice principal	47	43
SAT member 1	39	45
SAT member 2	35	19
SAT member 3	6	14
SAT member 4	44	41
SAT member 5	20	25
**Regular conversations with**		
Principal	75	100
Vice principal	73	98
SAT member 1	69	96
SAT member 2	74	98
SAT member 3	76	98
SAT member 4	74	98
SAT member 5	76	98
**Know more personally**		
Principal	98	100
Vice principal	98	100
SAT member 1	74	100
SAT member 2	98	100
SAT member 3	98	100
SAT member 4	97	100
SAT member 5	98	100
**Advice on a school matter**		
Principal	85	100
Vice principal	83	100
SAT member 1	82	95
SAT member 2	80	100
SAT member 3	82	96
SAT member 4	82	96
SAT member 5	82	96

2-step reach is the proportion of people in the total group (n = 50) who can be reached within one link of a person’s immediate personal ties.

SAT = CORE School Action Team member.

**Figure 1:**
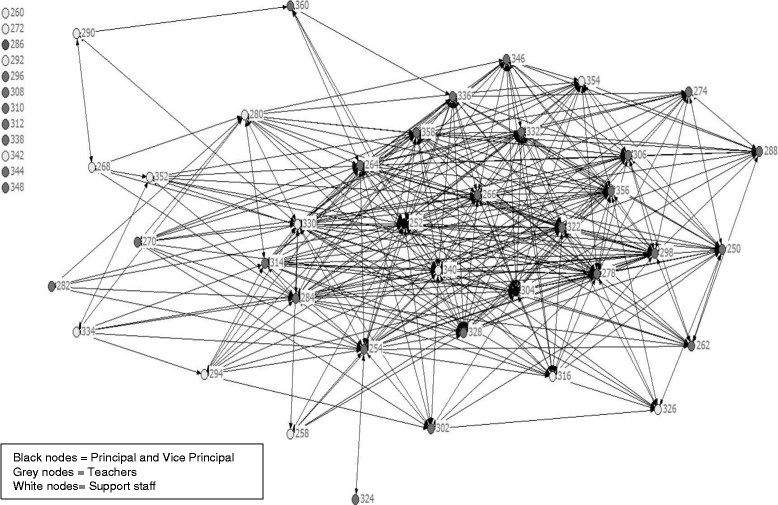
**Staff network of regular conversations at the start of CORE.**

**Figure 2:**
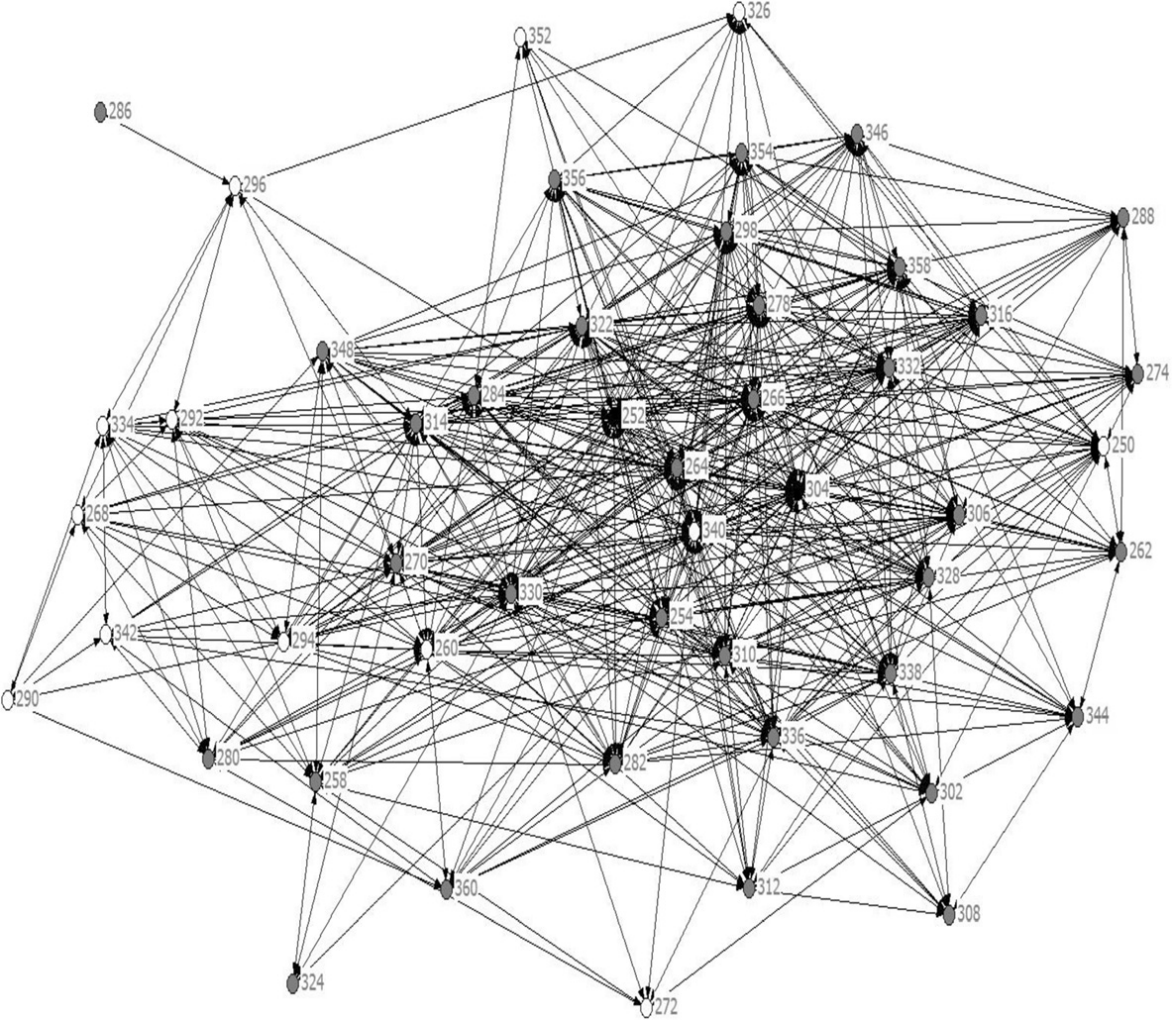
**Staff network of regular conversations after a year of CORE.**

Figures 1 and 2 illustrate the changes in the structure of the “regular conversations” network at school. At the beginning of CORE there were 12 disconnected people. Twelve months later all members of staff were connected.

## Discussion and conclusions

Overall, the results from CORE were similar to those of the Gatehouse Project, that is, with no significant change in students’ depressive symptoms, but change in a cluster of risk behaviours notably, drinking, unprotected sex and poor health among girls. Two aspects of the findings were different. Gatehouse showed no change on connection to school (the purported intermediary variable). By contrast we found a change in connection to school for girls, but not boys. Gatehouse showed an impact on risk behaviours, with no gender differences (L. Bond personal communication) whereas, we only showed significant changes in the health and risk behaviours in girls. Linked longitudinal data would have allowed us to test changes in school engagement at the individual level (i.e., to determine if those students who increased their engagement to school also decreased the risk behaviours). We chose instead to reassure students by using no ID codes, to confirm anonymity and potentially maximise honesty of responses. To investigate one possible mechanism of action, we turned to social network analysis to get a better understanding of the dynamics of change. The network analysis revealed that staff and teachers became significantly more interconnected.

The Gatehouse Project was a cluster randomised control trial. By contrast, this before-and-after study in a single school is a weaker design, lacking a comparison group and limited in its capacity for causal inference. The results are also vulnerable to the possibility that students may have reported their behaviour more positively after 2 years as a consequence of perceived social pressure to do so, particularly as CORE was so prominent in the school. But this interpretation is unlikely. First, social desirability bias, if it were present would likely have had more students describing their connection to school as strong, as this was the obvious focus of CORE. If social desirability were present it appears to only operate through girls. It is unclear why girls would feel more compelled than boys to offer socially desirable responses. Second, students were unlikely to be sensitive to the risk behaviours of interest because prevalence data from baseline, was never fed back (the school only received data about connection to school and feeling safe) and the risk factors of interest were ‘buried’ in an omnibus survey that also asked about things like physical activity, employment and connection to neighbourhood which did not alter in response to CORE.

Further, both the original Gatehouse Project, and its replication (CORE), made no mention of drinking, smoking, unsafe sex or drug use. The intervention was entirely and exclusively focused on making the school a better place for everyone. This is an important point. As a consequence of it being misunderstood, the Gatehouse Project results have been recently criticised [55]. That is, the charge has been made that decreases in substance use were the consequence of social desirability bias, because program elements aimed to alert students to such risk factors and were the focus of the curriculum [55]. This is not true. Risk factors were not part of the curriculum or a focus at all. Further, this replication case study illustrates a similar pattern in a different context (older students, different school system) as a result of the same strategy. That is, a holistic intervention to enrich the social environment, with students and staff as active agents in addressing problems appears to be risk protective, with no attention paid to the risk behaviours themselves.

However, the view of what happened within the CORE intervention remains partial. Schools that have undertaken similar projects produce qualitative accounts of changes in self-regard and pride, engagement and aspiration, and a sense of security and support [16] as well as changes in the culture, feel and everyday language of the school [56]. This study shows that social network analysis methods may also be a promising means to capture the dynamic of change processes in schools with staff and potentially in future with students also. It is a different approach to social environment measurement than those that involve self-appraisal of a person’s engagement, commitment or belonging [57]. We found statistically significant increases in the density of all five social relationships among staff. The changes in the social network structures paint a picture of staff becoming more cohesive and collaborative. This type of social environmental change with staff has not been observed with the social network analysis in whole school interventions previously, to our knowledge. Whether this pattern also occurred in the Gatehouse Project is unknown. In this study we can speculate that a more connected staff may have been more capable, engaged and responsive to student concerns. The social network data have no comparison group. So we do not know the extent to which increases in densities across time are natural, versus attributable to the change processes under study. We also cannot discount the possibility that staff gave answers that they felt would be socially acceptable. On the other hand, if these scenarios were true we would expect higher densities after a year of intervention and less discrimination among the relationships measured. In other words, inflated answers would have been applied in all categories if staff felt obliged to say they were mixing or interacting more, rather than the pattern that was observed, where some network densities (such as socialising with) showed a small increase but remained low across two years.

Much of the change in the social networks was attributed to the decision taken to abandon the usual practice of ‘retreat style’ full day professional development sessions for staff, in favour of weekly one hour sessions throughout the year. In this sense, the objective (to increase skills in teaching for emotional literacy) achieved both an educational and structural purpose. It created a weekly “check in” procedure across the school that kept the goal of whole school change at the forefront of people’s minds and a new way of working together. It was also where the CORE facilitator implemented strict rules about mixing people together. This may have been critical to the change process. The interviews with key informants undertaken for process monitoring purposes (data not reported here) placed high value on the creation of this new professional space enabling staff to work together for school improvement, noting that some ancilliary or support staff were motivated to attend the after school sessions even though they were not paid to come or compensated with time off. Members of the CORE school action team told us that the mixing of staff possibly transferred skills from staff who were comfortable mixing with students to those who were not. CORE encouraged staff to show that they cared, even just in small ways, such as noticing students in the hallway, smiling, and saying hello. Staff also confessed that they were embarrassed by the extent to which (until CORE) staff did not know each other.

Depressive symptoms in students was a key variable of interest in the Gatehouse project design and in this replication. Overall, there was little change in depressive symptoms over a 2 year time frame. Prevalence for girls dropped slightly from 17% to 15% (not statistically significant) and boys remained at 11%. The Gatehouse Project also showed no reduction in depressive symptoms as have other similar studies [58]. The reasons why these interventions have not shown an impact on depression are unclear. It may be that depression is more intractable than other health risk behaviours; that depressive symptoms remain but response to these feelings changes (e.g. do not self-medicate via drug use) and/or there are incompletely understood causal pathways [20]. That is, while alienation from school predicts risk behaviours and depressive symptoms, action to promote engagement reduces risk behaviours but not depression. Universal classroom-based methods to prevent depression have shown small but consistent effects [59]. It should be noted that CORE contained no formal social emotional learning curriculum as such. A review of whole school ethos-changing interventions found no evidence for effectiveness on depression, noting that it may be difficult to make the relevant environmental changes in complex organisations and to measure them effectively [60]. So while specific curriculum approaches produce small effects for both depression (and risk behaviours), the large effects for risk behaviours from whole school approaches still provide encouragement to see if similar gains could be made for depression, given that whole school approaches do not compete for class time and have the potential to deliver multiple benefits [61]. It may also be that whole school approaches affect emotional well being, rather than depression, and so effects are not being adequately captured.

The differential impact of CORE on risk behaviours according to gender is interesting. No gender effects have been reported to date by a similar Gatehouse replication program of work in the UK by Bonell and colleagues [16,62]. However, in the USA a whole school ethos building intervention combined with a curriculum targeted at prosocial behaviours and emotional literacy in inner-city African-American schools was more successful than a curriculum-only approach with boys’ risk behaviours only. Those authors argued that the effect in girls may have been missed (a measurement issue in not capturing the types of aggression that are more prominent in girls) [63]. The other explanation offered was that in very hostile contexts girls maintain aggressive behaviours to present a tough image to protect themselves [63]. Other researchers have also reported differential effects by gender for interventions targeting risk behaviours [64]. We searched the journal kept by the CORE facilitator, the key informant interview records and all other documentary records of the intervention’s unfolding and could find no evidence that CORE was targeted preferentially to girls or that girls responded or engaged differently in activities. On the other hand, we did not structure reporting on boy/girl involvement formally into our procedures and we would recommend that this be done more systematically in future. Future social network analytic methods among students could investigate differential impacts on boys and girls.

Regression to the mean is a possible explanation for the change in girls’ risk behaviours in our study. Concern about sexually transmitted disease rates prompted contact with the research team, and the largest risk reductions were seen in unprotected sex and poor self-rated health among girls. However, comparison of our data with a nationally representative prevalence survey of Canadian adolescents suggests that this was not the driver of the effects we observed. Nationally, the prevalence of unprotected sex for Grade 10 girls in Canada remained constant between 2003 and 2005 at 31% [37]. The prevalence of unprotected sex in our sample was 18% in 2003, lower than the national average, and it moved further from the national trend to only 8% in 2005. School connection for girls also improved during CORE. (In contrast, the national survey indicates drinking alcohol reduced in Canada from 2002 to 2006 for both boys and girls [37], and this fitted the trend in our own data).

This reduction in risk behaviours in the town’s only high school occurred at a time when social turbulence in the town itself (associated with the expansion of the meat packing industry) saw crime rates increase and case loads of child and family service agencies double [32]. Note again that the CORE intervention made no mention of sexual health in any program materials, the way the intervention was talked about, or in the hiring and briefings of project team members. We also found no found evidence of any alternative concurrent intervention or explanation for the CORE effects from project records. This would seem to support the conclusion that something protective happened at the high school during this time, which impacted girls at least, and unprotected sex may have been more sensitive and more responsive to a whole school ethos-focused intervention in this school. While the results of the project were fed back to the school, we regret that we did not take the opportunity to formally research and document the local interpretations and explanations staff and students might give for the effects observed. We suggest that this occur in future repetitions of the intervention.

Ever since the early attention drawn to this field by Rutter [65], researchers have been alert to the special role of school context seen in “school level effects”, meaning differences in risk behaviours and student outcomes that are attributable to the school, rather than to the compositional characteristics of the students themselves [66]. In spite of this, a large portion of school level preventive programs continue to focus on specific risk behaviours, transferring identical risk factor reduction curricular from one school to another [67] paying scant attention to differences in context that might call for a different response. Even researchers who have advanced understanding of the clustering of adolescent risk behaviours and the creation of normative and maladaptive trajectories over time, appear to abandon their own ecological thinking when it comes to prevention, by urgently calling for ‘health education’ programs to address risks [68]. Alternative interventions, of the type described here are not well known.

This replication study and work by others [16,62] represents an intervention research agenda which mirrors the epidemiological research which suggests that we may have greater gains in prevention in the future by understanding how risks cluster, rather than by pursuing pathways that attempt to theorise alcohol, drug use, sexual behaviour, mental health separately [68,69]. This intervention research agenda seeks to enhance protective factors at school and reduce the general susceptibility to health risk [70,71]. It seems likely, therefore, that depending on the nature of the context, different risk factors will be more or less amenable to reduction. These may depend on the different compositional characteristics of the students, the characteristics of the schools, the number, initial prevalence and distribution of the risk behaviours. A further contributing factor may be the interconnectivity of the staff, and potentially the students. Experimenting with different risk behaviours is common in adolescence and considered one way in which adolescents try to establish autonomy and independence [72,73]. The extent to which a young person is able to successfully ‘slough off’ a risk behaviour they have simply ‘tried on’ may be affected by the availability of approachable adults at school and ease of contact with a variety of role models and ways of thinking/behaving. It may not simply be that adolescents need at least one connection with a supportive adult at school [74]. The adults themselves may need to be interconnected, valued and mutually supported. This thinking is consistent with an intervention being an event in a (complex) system, the effect and sustainability of which depends on how the dynamic properties of the system are harnessed [75]. These complex interdependencies are something which future studies should try to assess. Replication studies (sometimes derided as “me too!” studies) may therefore prove vital in building a stronger understanding of how context drives health outcome, and how context can be acted upon to change health outcome. This will enhance the field’s capacity to move beyond theories about individual behaviour and cognition to more fully understand and theorise schools as social environments [76].

## Appendix

Student Perceptions of School Scale

The school engagement questionnaire comprises 28 questions drawn from a range of instruments (see below). The psychometrics of the scale were tested in a large Australian based school intervention, which used all 28 questions [58]. The response scale for all the questions except question 21 was: YES! (interpreted as emphatically yes) yes, no, NO! (interpreted as definitely no). For question 21 the response was: none, 1–2, about half, almost all.

Beyondblue Schools Research Initiative Project Initiative [58]

1. I am encouraged to express my own views in my class(es)
2. Most of the students in my class(es) enjoy being together
3. Most of the students in my class(es) are kind and helpful
4. Most other students accept me as I am
5. Students at this school are encouraged to take part in activities, programs and special events

Gatehouse Project Adolescent Health Survey [1,41]

6. I try hard in school
7. Doing well in school is important to me
8. Continuing or completing my education is important to me
9. There are lots of chances for students at my school to get involved in sports, clubs and other activities outside class
10. Teachers notice when students are doing a good job and let them know about it
11. At my school, students have a lot of chances to help decide and plan things like school activities, events and policies

Manitoba School Improvement Survey [44]

12. My teachers are fair in dealing with students
13. Student activities at this school offer something for everyone
14. Students have a say in decisions affecting them at this school

Psychological Sense of School Membership [42]

15. There’s at least one teacher or other adult in this school I can talk to if I have a problem
16. I feel very different from most other students here
17. I can really be myself at this school
18. Other students in this school take my opinions seriously

Quality of School Life [45]

19. I feel I can go to my teacher with the things that are on my mind
20. Most of my teachers really listen to what I have to say
21. Thinking of my teachers this term, I really like (all/most/half/one or two/none)

Patterns of Adaptive Learning Survey [43]

22. In this school, teachers believe all students can learn
23. In this school, students’ ideas are listened to and valued
24. In this school, teachers and students really trust one another
25. In this school, teachers treat students with respect
26. This school really cares about students as individuals
27. I feel I belong at this school
28. I feel like I am successful in this school

## Abbreviation

CORE: The creating opportunity for resilience and engagement project
